# Melatonin alleviates oxidative stress damage in mouse testes induced by bisphenol A

**DOI:** 10.3389/fcell.2024.1338828

**Published:** 2024-02-19

**Authors:** Qi Qi, Jiaxin Yang, Shuang Li, Jingjing Liu, Da Xu, Guoqing Wang, Lei Feng, Xiaoyan Pan

**Affiliations:** ^1^ Center for Reproductive Medicine, Jilin Medical University, Jilin, China; ^2^ School of Medical Technology, Beihua University, Jilin, China

**Keywords:** melatonin, bisphenol A, oxidative stress, testis, testosterone

## Abstract

We investigated the effect of melatonin on bisphenol A (BPA)-induced oxidative stress damage in testicular tissue and Leydig cells. Mice were gavaged with 50 mg/kg BPA for 30 days, and concurrently, were injected with melatonin (10 mg/kg and 20 mg/kg). Leydig cells were treated with 10 μmol/L of BPA and melatonin. The morphology and organ index of the testis and epididymis were observed and calculated. The sperm viability and density were determined. The expressions of melatonin receptor 1A and luteinizing hormone receptor, and the levels of malonaldehyde, antioxidant enzymes, glutathione, steroid hormone synthases, aromatase, luteinizing hormone, testosterone, and estradiol were measured. TUNEL assay was utilized to detect testicular cell apoptosis. The administration of melatonin at 20 mg/kg significantly improved the testicular index and epididymis index in mice treated with BPA. Additionally, melatonin promoted the development of seminiferous tubules in the testes. Furthermore, the treatment with 20 mg/kg melatonin significantly increased sperm viability and sperm density in mice, while also promoting the expressions of melatonin receptor 1A and luteinizing hormone receptor in Leydig cells of BPA-treated mice. Significantly, melatonin reduced the level of malonaldehyde in testicular tissue and increased the expression of antioxidant enzymes (superoxide dismutase 1, superoxide dismutase 2, and catalase) as well as the content of glutathione. Moreover, melatonin also reduced the number of apoptotic Leydig cells and spermatogonia, aromatase expression, and estradiol level, while increasing the expression of steroid hormone synthases (steroidogenic acute regulatory protein, cytochrome P450 family 17a1, cytochrome P450 17α-hydroxylase/20-lyase, and, 17β-hydroxysteroid dehydrogenase) and the level of testosterone. Melatonin exhibited significant potential in alleviating testicular oxidative stress damage caused by BPA. These beneficial effects may be attributed to melatonin’s ability to enhance the antioxidant capacity of testicular tissue, promote testosterone synthesis, and reduce testicular cell apoptosis.

## 1 Introduction

Bisphenol A (BPA) is a widely encountered environmental pollutant. It is a major raw material for polycarbonate and epoxy resins, used in the production of food/beverage containers, thermal receipts, dental sealants, and other items (Russo-Marie, 1986). Under high temperatures (such as in microwaves or dishwashers), and acidic or alkaline conditions, BPA monomers can dissociate from polymers and be released into the environment, posing potential exposure risks to humans (Russo-Marie, 1986). Research has found that BPA can cause severe male reproductive toxicity (Russo-Marie, 1986), resulting in compromised semen quality and damage to sperm DNA integrity (Snijder et al., 2013). Moreover, it can lead to reduced testosterone synthesis (Akingbemi et al., 2004; Roy et al., 2004; Meeker et al., 2010) and impaired supporting cell activity (Bindhumol et al., 2003; Fiorini et al., 2004; Salian et al., 2009).

Oxidative stress damage to testicular tissue caused by BPA is the main reason for disrupting the development of the male reproductive system (Pasqualotto et al., 2000). BPA can elevate reactive oxygen species (ROS) in sperm (Dobrzynska and Radzikowska, 2013; Liu et al., 2013), disrupting the antioxidant system in germ cells and causing impaired development and apoptosis of germ cells. Eliminating the excessive oxygen free radicals caused by BPA and enhancing the antioxidant capacity of testicular cells can alleviate oxidative stress damage to testicular cells and protect testicular spermatogenic function.

Melatonin (N-acetyl-5-methoxytryptamine) is a neuroendocrine hormone secreted by the pineal gland, which possesses strong antioxidant capabilities (Tan et al., 2007). This hormone can also be synthesized in the testes (Frungieri et al., 2017). Melatonin activates antioxidant enzymes and eliminates ROS (Amaral and Cipolla-Neto, 2018), thereby protecting the testes from inflammatory damage (Frungieri et al., 2017). Melatonin is both lipophilic and hydrophilic, allowing it to directly pass through cell membranes, thereby activating intracellular antioxidant pathways and regulating cell apoptosis (Claustrat and Leston, 2015; Li et al., 2018). Additionally, it can exert antioxidant functions by binding to melatonin receptors 1 and 2 on the cell membrane and interacting with G protein-coupled receptors (Samanta, 2020). Melatonin has been found to significantly alleviate testicular oxidative stress induced by the endocrine disruptor di(2-ethylhexyl) phthalate (Bahrami et al., 2018). The potential impact of BPA as an endocrine disruptor on testicular cells should not be underestimated, given its ability to induce oxidative stress damage. Due to the potent antioxidant properties of melatonin, we speculate that it may alleviate the oxidative stress induced by BPA in testicular cells and enhance male reproductive capability.

Herein, we investigated the potential role and mechanism of melatonin in mitigating testicular tissue oxidative stress caused by BPA. Our findings suggest that melatonin may serve as a novel therapeutic drug that can mitigate oxidative stress-induced damage in testicular cells and enhance male fertility.

## 2 Materials and methods

### 2.1 Study animals

Sexually mature Kunming male mice (n = 50; body weight: 35 g±2g; age: 2 months old) were purchased from Yisi Experimental Animal Technology Co., Ltd. (Changchun, China). The mice were housed in a temperature-controlled room (22°C ± 2°C) under a 12-h light/dark cycle. The study was carried out following the Declaration of Helsinki and approved by the Ethics Committee of Jilin Medical University (approval number: 2022-KJT016; date of approval: 2022-09-12). All animal experiments complied with the ARRIVE guidelines.

### 2.2 Preparation of BPA and melatonin

BPA (239658, Sigma-Aldrich, St. Louis, MO, USA) was dissolved in olive oil and the final concentration of the stock solution was 20 mg/mL. Melatonin (M5250, Sigma-Aldrich) was dissolved in a mixture of ethanol and normal saline (volume ratio 1:9) (Suwannakot et al., 2022). The final concentration of the stock solution was 8 mg/mL.

### 2.3 Animal grouping and treatment

The mice were randomly divided into five groups (n = 10), namely, the control group, BPA group, 20MT (melatonin) group, BPA+10MT group, and BPA+20MT group. The mice in the BPA group, BPA+10MT group, and BPA+20MT group were administered 50 mg/kg BPA daily by gavage. This dose was considered the lowest-observed-adverse-effect level (Rahman et al., 2017). The mice in the control group and the 20MT group were gavaged with an equivalent volume of olive oil daily, while the mice in the BPA+10MT group received an intraperitoneal injection of 10 mg/kg melatonin every other day. The mice in the 20MT group and BPA+20MT group were injected with 20 mg/kg melatonin every other day (Zencirci et al., 2010). The mice in the control group and BPA group were injected with an equivalent volume of normal saline every other day. The mice in all five groups were intervened for 30 days.

### 2.4 Sample collection

After the intervention, the body weight of mice was measured. Then, the mice were sacrificed through cervical dislocation. The testes and epididymis were harvested, with subsequent removal of surrounding fatty tissues. The testes and epididymis were individually weighed, and their morphology was documented through photographs. The testis index (ratio of testis weight to body weight) and the epididymis index (ratio of epididymis weight to body weight) were calculated. Sperm viability in the cauda epididymis was evaluated, followed by density analysis. The right testis of the mice was preserved in Bouin’s solution (BL-GO16, SenBeiJia Biological Technology Co., Ltd., Nanjing, China), while the left testis was frozen at −80°C for future experiments.

### 2.5 Leydig cell culture and treatment

The mouse Leydig cell line TM3 was obtained from Cell Bank (CL-0234, Wuhan Punosai Life Technology Co., LTD, China). These Leydig cells were cultured in DMEM/F12 medium (8123462, Gibco, Grand Land, NY, USA) supplemented with 10% fetal bovine serum (BL201A, Biosharp, Hefei, China) and 1% penicillin-streptomycin (P1400, Solarbio, Beijing, China) and incubated at 37°C with 5% CO2. Cells were passaged at 80%–90% confluence every 2-3 days. BPA (239658, Sigma-Aldrich) and melatonin (M5250, Sigma-Aldrich) were dissolved in dimethyl sulfoxide (DMSO) to prepare stock solutions at concentrations of 75 mmol/L and 0.1 mol/L, respectively. The stock solutions were then diluted with culture medium to achieve final culture concentrations of 10 μmol/L for both BPA and melatonin (Goncalves et al., 2018; Zhang et al., 2022).

Leydig cells were divided into three groups: control, BPA, and BPA + MT. The control group cells were cultured in a normal medium for 24 h. The BPA group cells were treated with 10 μmol/L BPA for 24 h (Goncalves et al., 2018). The BPA + MT group cells were simultaneously treated with 10 μmol/L BPA and 10 μmol/L melatonin for 24 h (Zhang et al., 2022). The final concentration of DMSO in the BPA group was 0.013%, while in the BPA + MT group, it was 0.023%. It has been demonstrated that 0.1% DMSO has no toxic effect on Leydig cells (Zhang et al., 2022). Therefore, a DMSO control group was not included.

### 2.6 HE staining

The mouse testes were fixed in Bouin’s solution for 24 h, followed by dehydration in a series of ethanol solutions (70%, 80%, 90%, 95%, 100% I, and 100% II) for 1 h each. Subsequently, the tissues were subjected to xylene treatment for 15 min and then embedded in paraffin. The embedded testicular tissues were cut into 5 μm sections. The sections were then deparaffinized with xylene and subjected to a gradient ethanol treatment. After staining with hematoxylin (S0152, Beijing Bioss Biotechnology, China) for 4 min and eosin (C145380, Sinoreagent, Ningbo, China) for 1 min, the sections were mounted with neutral resin. The tissue structure of the testes was observed and captured using an Olympus BX53 microscope (Tokyo, China). The thickness of the germinal epithelium was analyzed using ImageJ software (Java 1.8.0, National Institutes of Health, Bethesda, MD, USA) (Zhang et al., 2018).

### 2.7 Quantification of sperm viability and density

The mouse cauda epididymis was placed in 1 mL of preheated phosphate buffer saline (PBS). Subsequently, the cauda epididymis was punctured using a 26G needle and then incubated at 37°C for 15 min. After incubation, 150 μL of sperm suspension was extracted and combined with 10 μL of 1% eosin. The resulting mixture was utilized to prepare sperm smears, followed by microscopic observation of stained sperm heads using an Olympus IX83 microscope. Dead sperm with compromised plasma membranes demonstrated uptake of the eosin dye, appearing red, while live sperm with intact plasma membranes remained unstained. Live and dead sperm counts were obtained from 5 mice to determine the sperm viability.

For sperm density analysis, the post-incubation sperm suspension was diluted at a 1:10 ratio with 3% NaCl solution. The diluted sperm suspension (10 μL) was used to enumerate sperm on a hemocytometer for sperm density determination.

### 2.8 Immunohistochemistry

The testicular tissue sections were deparaffinized with xylene and then subjected to gradient alcohol treatment. After that, the sections were incubated with 3% hydrogen peroxide (H2O2) for 10 min to inactivate endogenous peroxidases. The sections were then subjected to antigen retrieval by boiling in ethylene diamine tetraacetic acid antigen retrieval buffer (RA0023, Beijing Bioss Biotechnology) for 10 min. Subsequently, the sections were blocked with 5% bovine serum albumin (BSA) at 37°C for 30 min, followed by overnight incubation at 4°C with a rabbit polyclonal antibody against melatonin receptor 1A (MTNR1A) (A13030, ABclonal, Boston, MA, USA, 1:200 dilution). After rinsing three times with PBS, the sections were incubated at 37°C for 30 min with biotinylated goat anti-rabbit IgG (BA1006, Wuhan Boster Biological Technology, China). After washing with PBS three times, the sections were incubated with streptavidin-biotin complex (SABC) (SA1022, Wuhan Boster Biological Technology, China) at 37°C for 30 min, followed by three PBS washes. Diaminobenzidine (AR1027, Wuhan Boster Biological Technology) was used for color development, and counterstaining was performed with hematoxylin. The expression intensity of MTNR1A was observed under an Olympus BX53 microscope and analyzed using ImageJ software (Java 1.8.0, National Institutes of Health, Bethesda, MD, USA).

### 2.9 Western blotting

The proteins were extracted from the testicular tissues after lysis with Radio-Immunoprecipitation Assay buffer (containing 1% phenyl methane sulfonyl fluoride) (P0013B and ST505, Beyotime). The total protein concentration was determined using the bicinchoninic acid protein assay kit (P0015S, Beyotime). An equal amount of protein was separated on 8% SDS-PAGE and transferred to polyvinylidene difluoride (PVDF) membranes (IPVH00010, Millipore, Billerica, MA, USA). After blocking with 5% skim milk for 1 h, the PVDF membrane was incubated with rabbit-derived anti-MTNR1A (A13030, ABclonal) and anti-β-actin (AC026, ABclonal) primary antibodies overnight at 4°C. After washing, the PVDF membrane was incubated at room temperature with goat anti-rabbit horseradish peroxidase-conjugated secondary antibody (A0208, Beyotime) for 2 h, followed by enhanced chemiluminescence detection. The membrane was then scanned using a ChemiDOC XRS + imaging system (Bio-Rad Laboratories, Hercules, CA, USA). ImageJ software was used to analyze the relative expression level of MTNR1A.

### 2.10 Malondialdehyde (MDA) detection

The testicular tissue (1 g) was homogenized in 9 mL of PBS buffer supplemented with a protease inhibitor cocktail (EDTA-Free) (K1007, APExBIO, Houston, TX, USA). The resulting homogenate was then centrifuged at 2000–3000rpm and 4°C for 20 min, and the supernatant was subsequently collected. The protein concentration in the samples was determined using the Bradford Protein Assay Kit (P0006, Beyotime, Shanghai, China). The MDA content in the samples was measured using the MDA Assay Kit (S0131S, Beyotime). At high temperatures, MDA can react with thiobarbituric acid (TBA) to form a red MDA-TBA adduct, which exhibits maximum absorption at 535 nm. The absorbance of the MDA-TBA adduct was measured at a wavelength of 535 nm using a microplate reader (CMax Plus, Molecular Devices (Shanghai), China). The MDA concentration was calculated by dividing the measured MDA content by the protein content of the sample.

### 2.11 ELISA

The supernatant of homogenized testicular tissue and the culture supernatant of Leydig cells were both collected. ELISA detection kits (Shanghai Yuanju Biotechnology Center, China) were utilized to determine the levels of superoxide dismutase 1 (SOD1) (YJ037856), superoxide dismutase 2 (SOD2) (YJ058668), catalase (CAT) (YJ037752), glutathione (GSH) (YJ063305), steroidogenic acute regulatory protein (StAR) (YJ963054), cytochrome P450 family 17a1 (CYP11A1) (YJ212183), cytochrome P450 17α-hydroxylase/20-lyase (CYP17A1) (YJ053990), 17β-hydroxysteroid dehydrogenase (17β-HSD) (YJ930573), cytochrome P450 family 19 (CYP19) (YJ202547), luteinizing hormone (LH) (YJ063366), testosterone (YJ001948) and 17beta-estradiol (E2) (YJ001962). The absorbance readings were obtained at a wavelength of 450 nm using a CMax Plus microplate reader (Molecular Devices (Shanghai)). Standard curves were constructed based on the concentration and absorbance values of the standard samples. Subsequently, the levels of SOD1, SOD2, CAT, GSH, StAR, CYP11A1, CYP17A1, 17β-HSD, CYP19, LH, testosterone, and E2 were determined by referencing the standard curves.

### 2.12 TUNEL assay

The TUNEL cell apoptosis assay kit (MK1024, Wuhan Boster Biological Technology) was used to detect cell apoptosis in testis tissue sections. In detail, the sections were subjected to deparaffinization and gradient ethanol treatment. Leydig cells were fixed with 4% paraformaldehyde. After digestion with proteinase at 37°C for 10 min, the sections or cells were then incubated with terminal deoxynucleotidyl transferase and digoxigenin-labeled dUTP (DIG-dUTP) at 37°C for 2 h, marking the 3′-OH end of fragmented DNA with DIG-dUTP. The sections or cells were blocked with 5% BSA blocking solution at room temperature for 30 min. Subsequently, the sections or cells were incubated with biotinylated digoxigenin antibody (diluted with SABC) at 37°C for 30 min, followed by washing three times, each time for 5 min. Additionally, the sections or cells were incubated with SABC-FITC at 37°C for 30 min, followed by 2-(4-Amidinophenyl)-6-indolecarbamidine dihydrochloride (C1005, Beyotime) staining at room temperature for 5 min. After staining, the tissue sections or cells were sealed with an anti-quenching mounting medium. Intestinal tissue was used as a positive control. The stained tissues or cells were observed under an Olympus BX53 microscope, and TUNEL-positive cells emitted green fluorescence. Three mice were selected from each group, and three sections of each mouse were stained. For Leydig cells, three slices of each group were assessed. A total of five randomly selected fields were analyzed, and the number of apoptotic cells in each field was quantified. The TUNEL-positive cell rate was calculated by the formula of (TUNEL-positive cell count)/(total cell count in the field of view) × 100%.

### 2.13 Immunofluorescence staining

The testicular tissue sections were deparaffinized and treated with gradient ethanol. Then, they were then subjected to antigen retrieval in ethylene diamine tetraacetic acid antigen retrieval buffer (RA0023, Beijing Bioss Biotechnology) for 10 min. After blocking with 5% BSA at 37°C for 30 min, the tissue sections were incubated with rabbit polyclonal antibody against luteinizing hormone receptor (LHR) (A13030, ABclonal, 1:400) at 4°C overnight. After washing, the sections were incubated with FITC-labeled goat anti-rabbit secondary antibody (A0562, Beyotime, Shanghai, China) at 37°C in the dark for 1 h. The cell nuclei were stained with 2-(4-Amidinophenyl)-6-indolecarbamidine dihydrochloride for 5 min, followed by slide sealing with an anti-fluorescence quencher. The regions expressing LHR exhibited green fluorescence under an Olympus BX53 microscope. The average fluorescence intensity of LHR expression was analyzed using ImageJ software. Three mice were analyzed in each group, with three slides per mouse, and the average fluorescence intensity of LHR in five random fields was analyzed.

### 2.14 Statistical methods

The experimental data were statistically analyzed using SPSS 26.0 software. The data are presented as mean ± standard deviation. The one-way ANOVA and LSD *post hoc* test were used for multiple group comparisons. *p* < 0.05 was considered statistically significant.

## 3 Results

### 3.1 Melatonin alleviates the inhibitory effect of BPA on the morphology and organ index of the testis and epididymis

**FIGURE 1:**
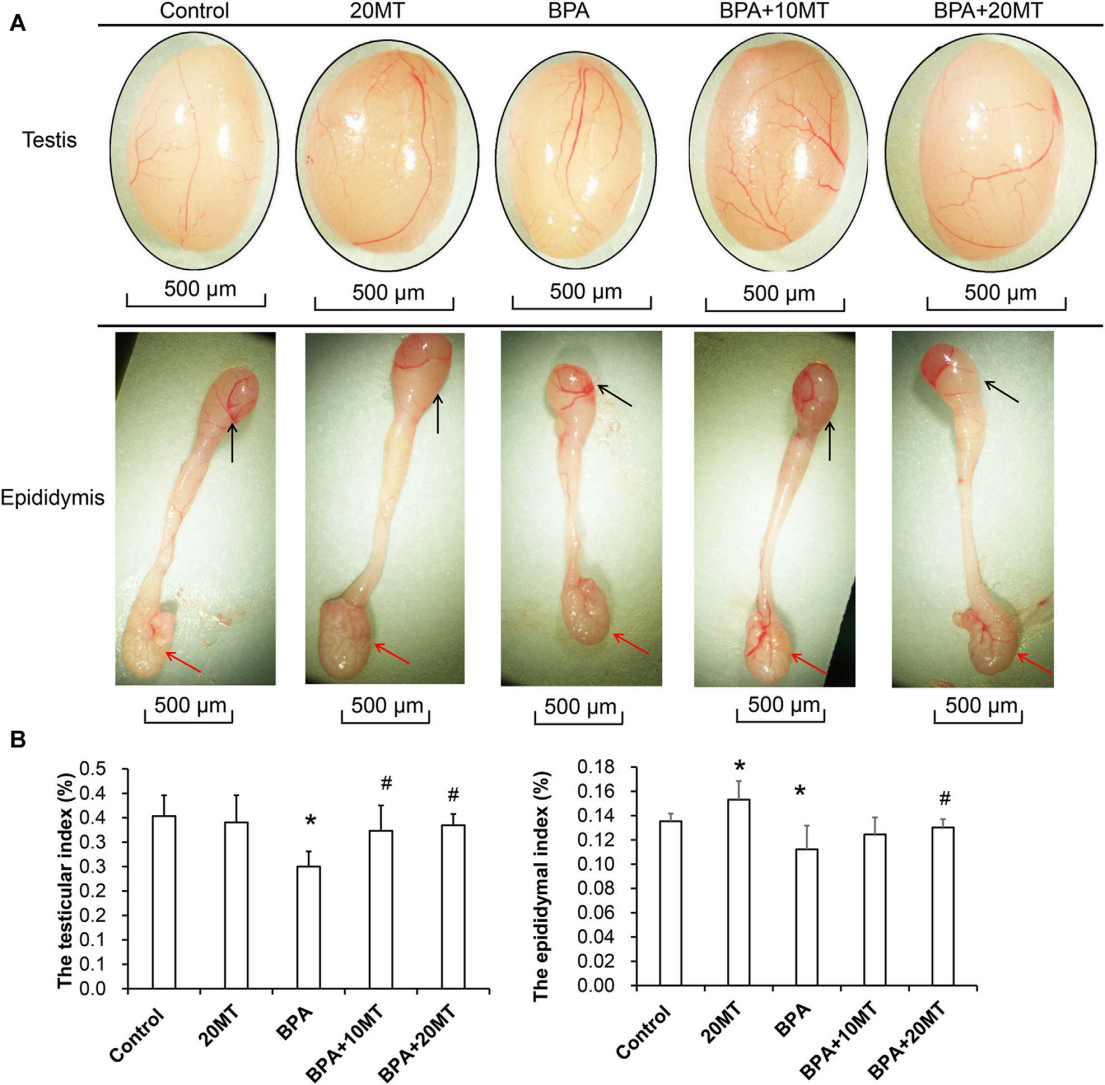
Analysis of testicular and epididymal morphology and organ index. **(A)** The gross morphology of mouse testes and epididymides. Black arrows indicate the epididymal head, while red arrows indicate the epididymal tail. **(B)** The testis index (ratio of testis weight to body weight) and the epididymis index (ratio of epididymis weight to body weight) (N = 5). Compared with the control group, ^*^ *p* < 0.05; compared with the BPA group, ^#^ *p* < 0.05.

After 30 days of intervention, the testes and epididymides of mice in each group were collected. Based on visual observation, the testes of mice in the BPA group exhibited a size reduction, while the length of the epididymides and the size of the epididymal tail were also markedly smaller (Figure 1A). In the 20MT group, the heads and tails of the epididymides were considerably larger. The morphological changes in the other groups were not obvious. The weights of one side of the testes and epididymides were measured, and the testicular index or epididymal index was calculated (Figure 1B). Statistically, the testicular index and epididymal index in the BPA group were significantly lower than those in the control group (*p* < 0.05). The testicular index of the 20MT group, BPA+10MT group, and BPA+20MT group did not show significant differences compared to the control group (*p* > 0.05), while the epididymal index of the 20MT group was significantly higher than that of the control group (*p* < 0.05). The testicular index of the BPA+10MT group and BPA+20MT group were significantly higher than that of the BPA group (*p* < 0.05), and there were no significant differences in the epididymal index between the BPA+10MT group and BPA group (*p* > 0.05). Additionally, there were no significant differences in the testicular index and epididymal index between the BPA+10MT group and the BPA+20MT group (*p* > 0.05). Consequently, melatonin (20 mg/kg) may ameliorate the inhibitory effects of BPA on the testes and epididymides.

### 3.2 Melatonin mitigates the damage caused by BPA to the seminiferous tubules in mice

**FIGURE 2:**
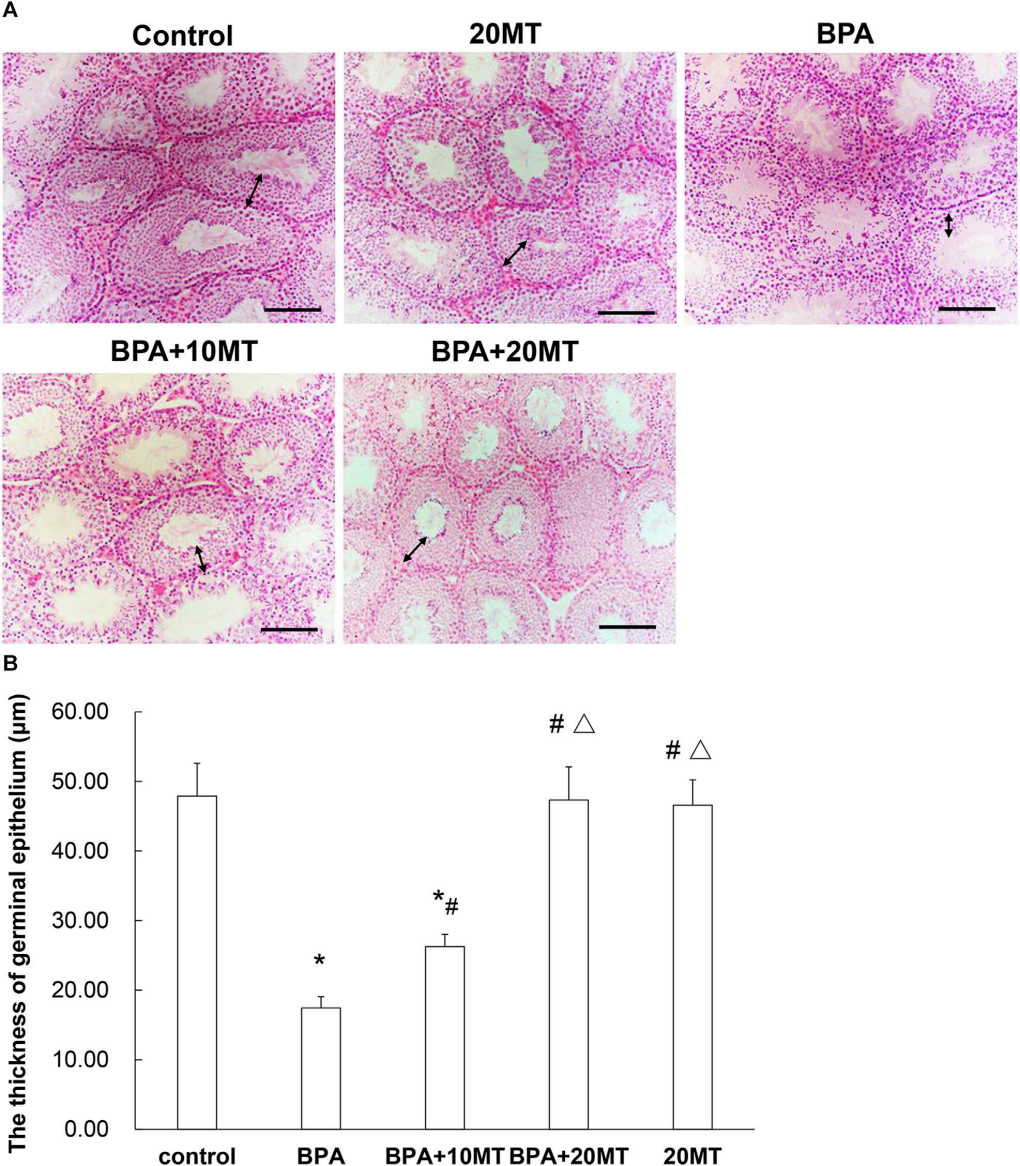
HE staining of mouse testicular tissue. **(A)** HE staining images of the control group, 20MT group, BPA group, BPA+10MT group, and BPA+20MT group are presented (Scale = 100 μm). **(B)** The thickness of germinal epithelium was measured (N = 5). The bidirectional arrows indicate the thickness of the germinal epithelium. Compared with the control group, ^*^ *p* < 0.05; compared with the BPA group, ^#^ *p* < 0.05, compared with the BPA+10MT group, ^△^ *p* < 0.05.

HE staining revealed that the epithelium of seminiferous tubules in the BPA group showed thinning, reduced germ cell count, disorganized arrangement of germ cells, and few sperm in the lumen, along with the presence of sloughed-off germ epithelium (Figure 2A). In the BPA+10MT group, there was a significant increase in the thickness of the germinal epithelium compared to the BPA group (Figure 2B). However, it remained thinner compared to the control group, 20MT group, and BPA+20MT group. The control group, 20MT group, and BPA+20MT group exhibited significantly thicker germinal epithelium than the BPA group, with germ cells exhibiting an organized arrangement, forming clusters of spermatozoa in the lumen, and no sloughed-off germ epithelium was observed (Figure 2). Thus, melatonin (20 mg/kg) can alleviate the damage caused by BPA to the seminiferous tubules in mice.

### 3.3 Melatonin improves the sperm viability and sperm density of mice treated with BPA

**FIGURE 3:**
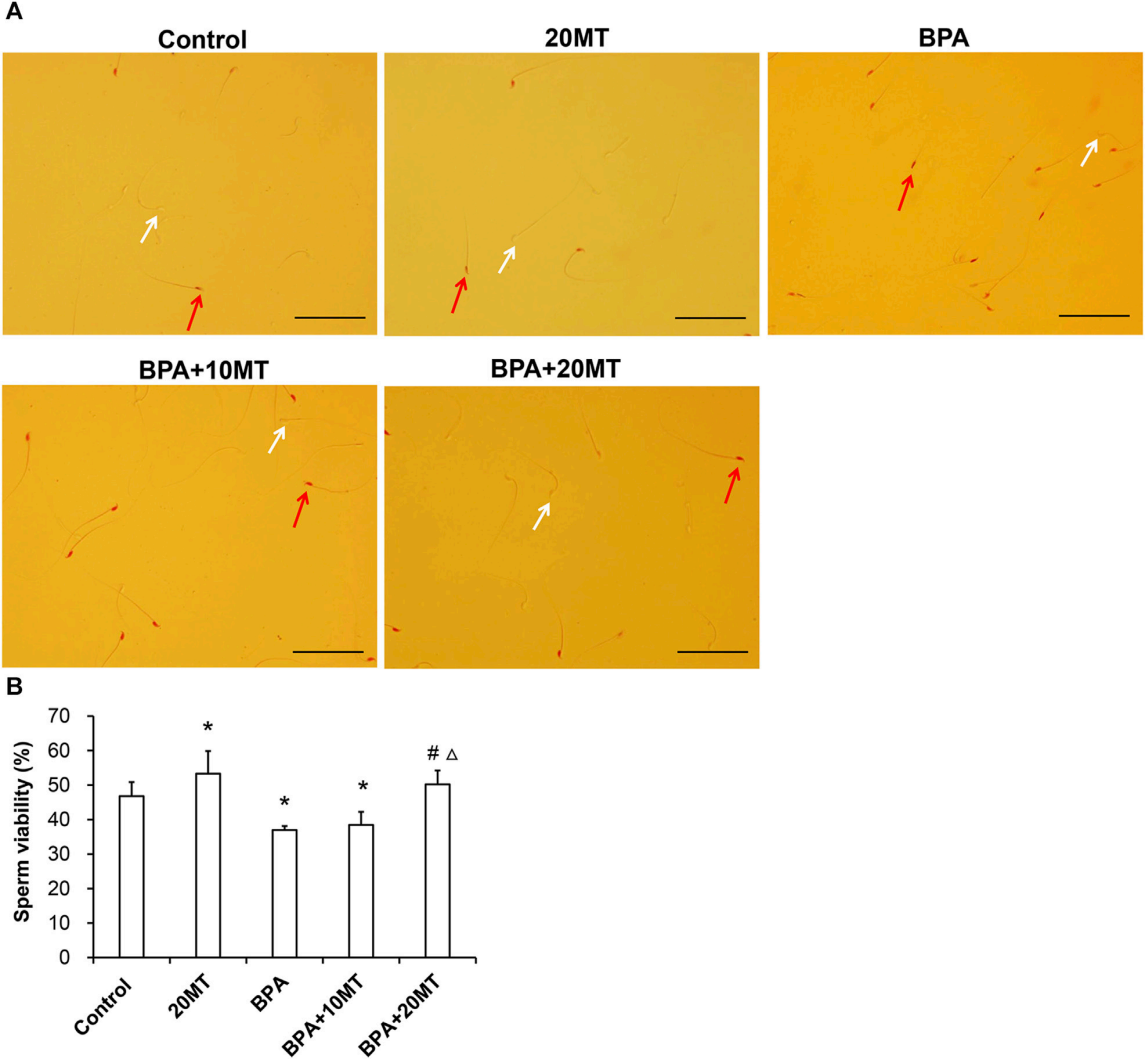
Assessment of viability of epididymal sperm in mice. **(A)** Sperm from the control group, 20MT group, BPA group, BPA+10MT group, and BPA+20MT group after eosin staining. White arrows indicate live sperm and red arrows indicate dead sperm (Scale = 100 μm). **(B)** Statistical analysis of the sperm viability in the epididymal tail of mice in each group (N = 5). Compared with the control group, ^*^ *p* < 0.05; compared with the BPA group, ^#^ *p* < 0.05, compared with the BPA+10MT group, ^△^ *p* < 0.05.

The cauda epididymis was collected and incubated (Figure 3A). The BPA group had a higher count of dead sperm and a lower count of live sperm. After melatonin intervention, there was a decrease in the number of dead sperm and an increase in the number of live sperm. Statistically, the sperm viability in the BPA group and BPA+10MT group was significantly lower than that of the control group (*p* < 0.05), while the sperm viability in the 20MT group was significantly higher than that of the control group (*p* < 0.05) (Figure 3B). There was no significant difference in sperm viability between the BPA+20MT group and the control group. The sperm viability of mice in the BPA group and BPA+10MT group showed no significant difference (*p* > 0.05), but the sperm viability of mice in the BPA+20MT group was significantly higher than that of the BPA group and the BPA+10MT group (*p* < 0.05). Hence, melatonin (20 mg/kg) may alleviate the decrease in sperm viability caused by BPA.

**FIGURE 4:**
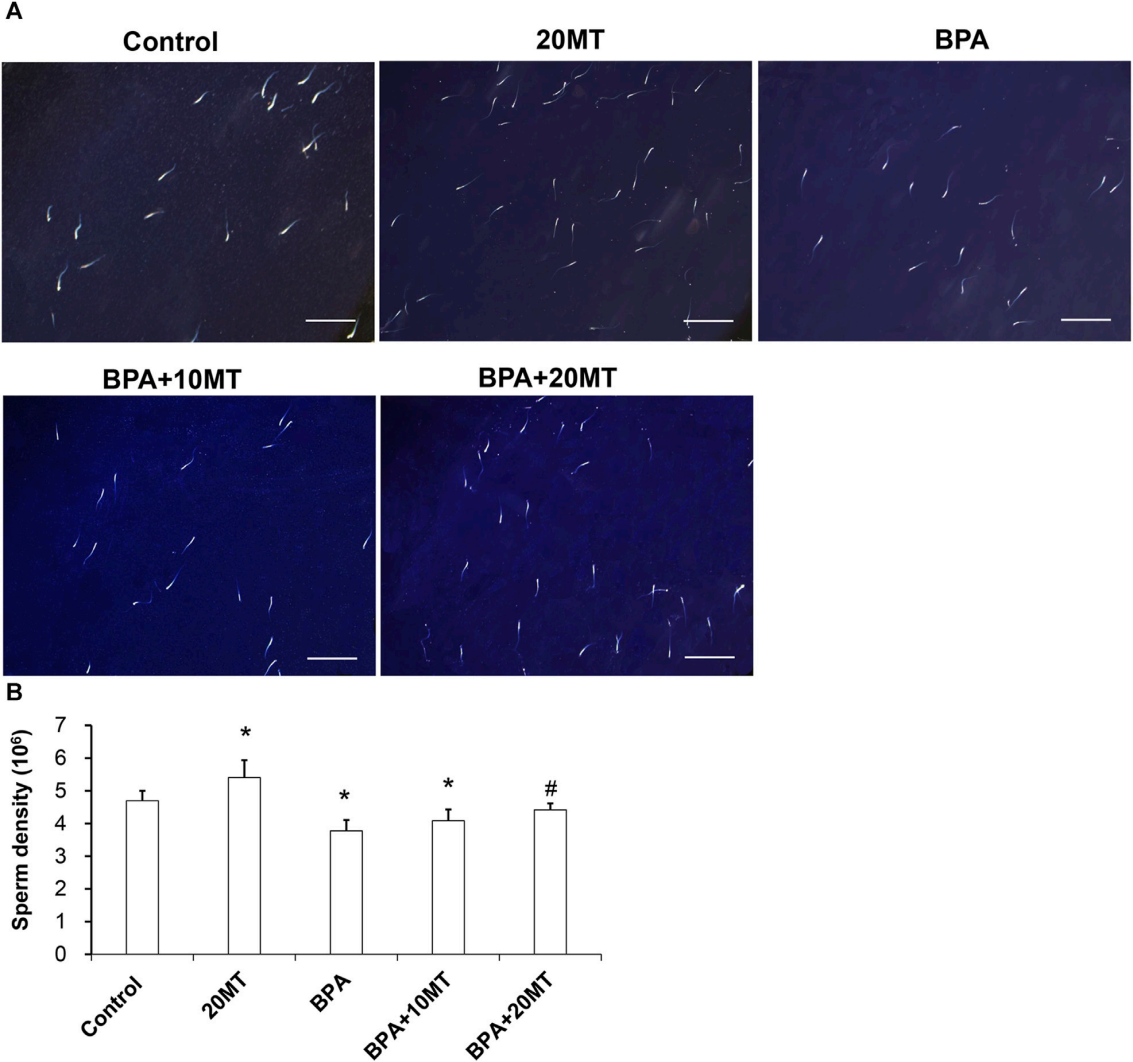
Analysis of epididymal sperm density in mice. **(A)** Sperm from the control group, 20MT group, BPA group, BPA+10MT group, and BPA+20MT group was observed under a microscope (Scale = 200 μm). **(B)** Statistical analysis of sperm density in each group of mice (N = 5). Compared with the control group, ^*^ *p* < 0.05; compared with the BPA group, ^#^ *p* < 0.05.

Sperm from each group was observed under a microscope (Figure 4A). The sperm count in the 20MT group was higher than the control group, while the BPA group and BPA+10MT group showed a decreasing trend in sperm count compared to the control group. The sperm count in the BPA+20MT group was similar to that of the control group. The sperm density was quantified by counting the sperms on the glass slide (Figure 4B). The epididymal sperm density was significantly higher in the 20MT group compared to that in the control group (*p* < 0.05). However, the BPA group and BPA+10MT group had significantly lower epididymal sperm density (*p* < 0.05). No significant difference in epididymal sperm density was observed between the BPA group and the BPA+10MT group (*p* > 0.05). In contrast, the BPA+20MT group exhibited a significantly higher epididymal sperm density compared to the BPA group and BPA+10MT group (*p* < 0.05). This indicates that melatonin (20 mg/kg) could mitigate the decrease in mouse sperm density induced by BPA.

The above results suggest that melatonin (20 mg/kg) may not have any detrimental effects on the function of normal testicular and epididymal tissues. It effectively alleviates the damage to the testes and epididymis caused by BPA and improves both the viability and density of mouse sperm. Hence, in the subsequent experiments, we selected melatonin (20 mg/kg) for investigation.

### 3.4 Melatonin enhances the expression of MTNR1A in mouse leydig cells treated with BPA

**FIGURE 5:**
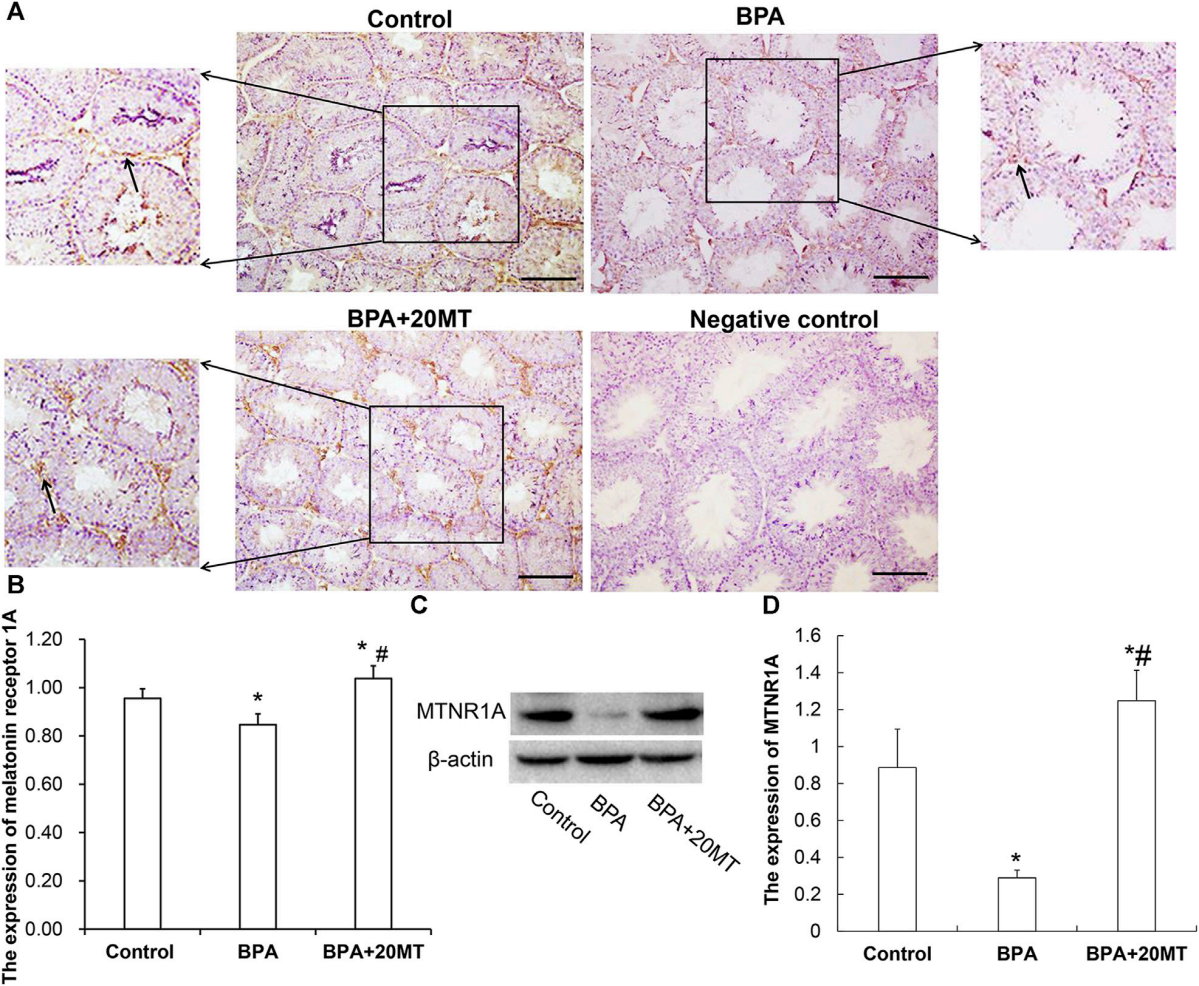
Expression of MTNR1A in mouse testicular tissue. **(A)** Immunohistochemical staining of MTNR1A in mouse testicular tissue (Scale bar = 100 μm). The black arrow indicates the expression of MTNR1A. **(B)** Expression of MTNR1A in mouse testicular tissue analyzed by grayscale value (N = 3). **(C)** Western blotting analysis of MTNR1A expression in mouse testicular tissue. **(D)** Relative level of MTNR1A expression (N = 3). Compared with the control group, ^*^ *p* < 0.05; compared with the BPA group, ^#^ *p* < 0.05.

To investigate the mechanism by which melatonin protects mouse testicular cells, we detected the expression of MTNR1A in mouse testicular tissue using immunohistochemistry and western blotting. MTNR1A was mainly expressed on the membrane of Leydig cells (Figure 5A). The Leydig cell membranes in the control group and the BPA+20MT group were stained more intensely, indicating a higher amount of MTNR1A expression. On the other hand, the Leydig cell membranes in the BPA group were stained less intensely, suggesting a lower amount of MTNR1A expression. Statistically, the expression of MTNR1A in the BPA group was significantly lower than that in the control group (Figure 5B). However, the expression of MTNR1A in the BPA+20MT group was significantly higher than that in the BPA group and control group (Figure 5B). Western blotting analysis of MTNR1A yielded consistent results with immunohistochemistry (Figures 5C,D). This data showed that melatonin (20 mg/kg) significantly increased the expression of MTNR1A on the membrane of Leydig cells.

### 3.5 Melatonin enhances the antioxidant capacity of mouse testicular tissue treated with BPA

**FIGURE 6:**
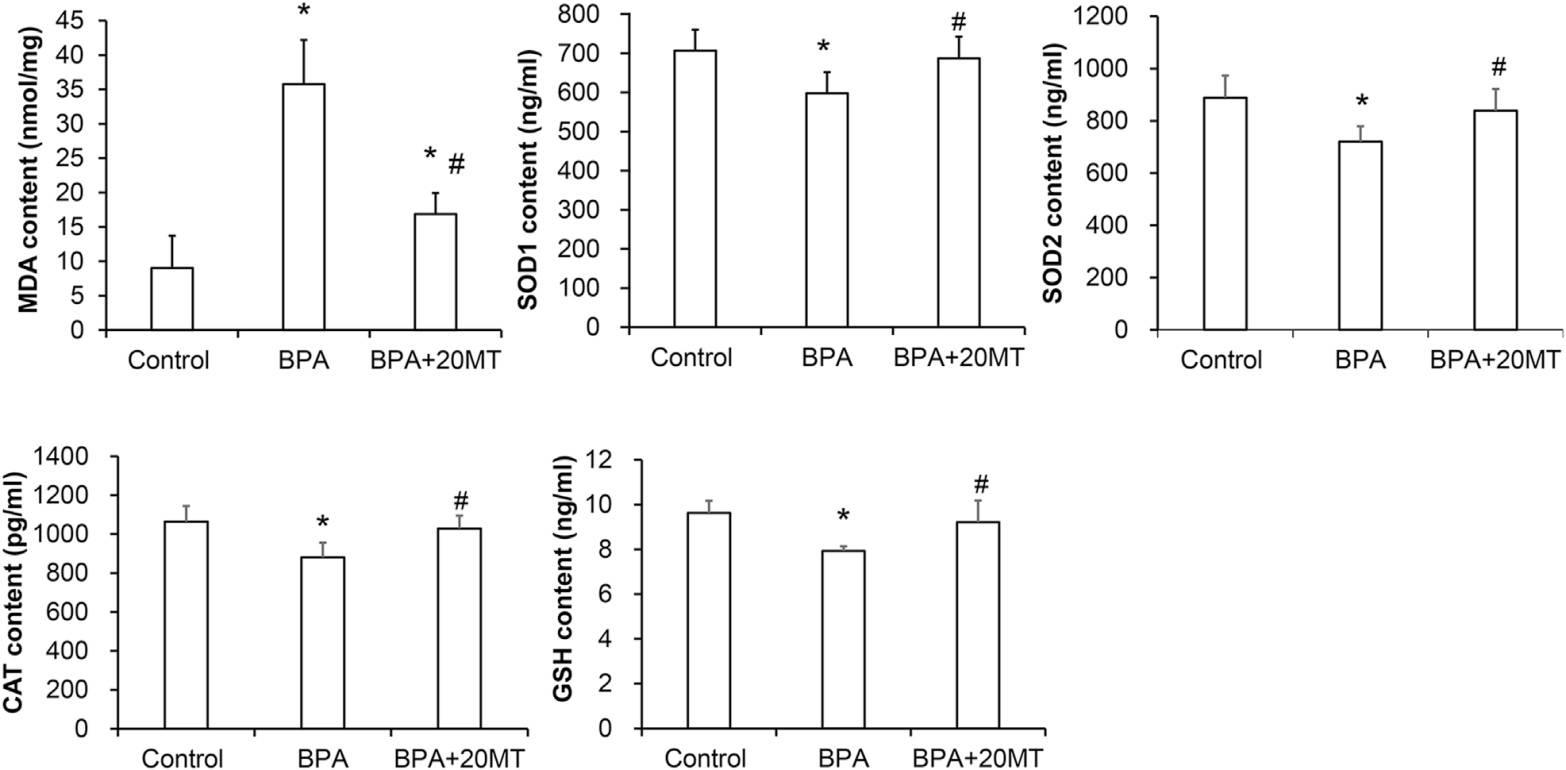
Assessment of antioxidant capacity in mouse testicular tissues. The contents of MDA, SOD1, SOD2, CAT, and GSH in mouse testicular tissues were measured (N = 5). Compared with the control group, ^*^ *p* < 0.05; compared with the BPA group, ^#^ *p* < 0.05.

To determine the oxidative damage of BPA on testicular tissue and the antioxidant effect of melatonin on testicular tissue, we detected the levels of MDA, SOD1, SOD2, CAT, and GSH in testicular tissue using the MDA assay kit and ELISA (Figure 6). The levels of MDA in the BPA group and BPA+20MT group were significantly higher than those in the control group (*p* < 0.05), but the levels of MDA in the BPA+20MT group were significantly lower than those in the BPA group (*p* < 0.05), indicating that melatonin (20 mg/kg) significantly reduces the lipid peroxidation in testicular tissue. The levels of SOD1, SOD2, CAT, and GSH in the BPA group were significantly lower than those in the control group (*p* < 0.05), while the levels of these enzymes in the BPA+20MT group showed no significant difference compared to the control group but were significantly higher than those in the BPA group (*p* < 0.05). This suggests that melatonin (20 mg/kg) could significantly increase the levels of antioxidant enzymes in testicular tissue.

### 3.6 Melatonin reduces apoptosis in mouse testicular cells treated with BPA

**FIGURE 7:**
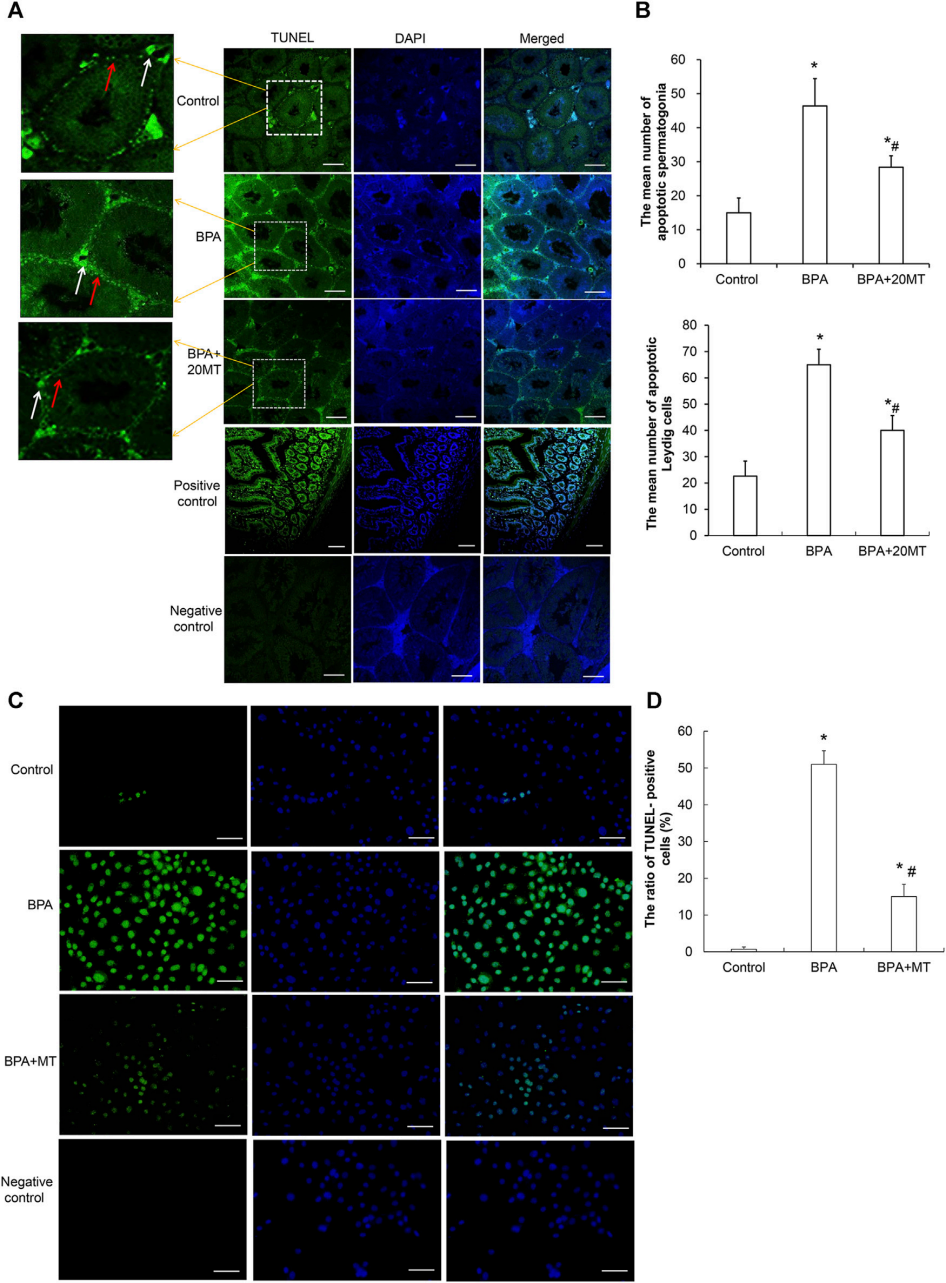
Detection of cell apoptosis in mouse testicular tissue and Leydig cells. **(A)** Cell apoptosis in mouse testicular tissue was detected using the TUNEL assay. Cells showing green fluorescence represent apoptotic cells (Scale = 100 μm). The white arrow indicates apoptotic Leydig cells. The red arrow indicates apoptotic spermatogonia. **(B)** The number of apoptotic cells was statistically analyzed in each group (N = 3). **(C)** The apoptosis of Leydig was determined using a TUNEL assay (Scale = 50 μm). **(D)** The ratio of TUNEL-positive cells was compared (N = 5). Compared with the control group, ^*^ *p* < 0.05; compared with the BPA group, ^#^ *p* < 0.05.

To examine the effects of BPA and melatonin on testicular cell apoptosis, we performed a TUNEL assay. BPA primarily induced apoptosis in spermatogonial cells and Leydig cells (Figure 7A). The control group and BPA+20MT group exhibited less green fluorescence, indicating less apoptosis in testicular cells. In contrast, the BPA group showed an increase in green fluorescence, especially in the interstitial region, suggesting an increase in apoptosis of germ cells and Leydig cells, with a predominant effect on Leydig cell apoptosis. The average number of apoptotic spermatogonial cells and Leydig cells in the seminiferous tubules of the BPA group was significantly higher than that of the control group and the BPA+20MT group (*p* < 0.05) (Figure 7B). Meanwhile, this number in the BPA+20MT group was significantly higher than that in the control group but significantly lower than in the BPA group (*p* < 0.05) (Figure 7B). Additionally, the TUNEL assay of Leydig cells revealed that the ratio of TUNEL-positive cells was significantly elevated by BPA, while it was significantly reduced by melatonin (*p* < 0.05) (Figures 7C, D). Therefore, melatonin may effectively alleviate testicular cell apoptosis induced by BPA.

### 3.7 Melatonin increases the expression of steroid hormone-synthesizing enzymes in the testicular tissue and leydig cells and decreases the expression of aromatase in the testicular tissue

**FIGURE 8:**
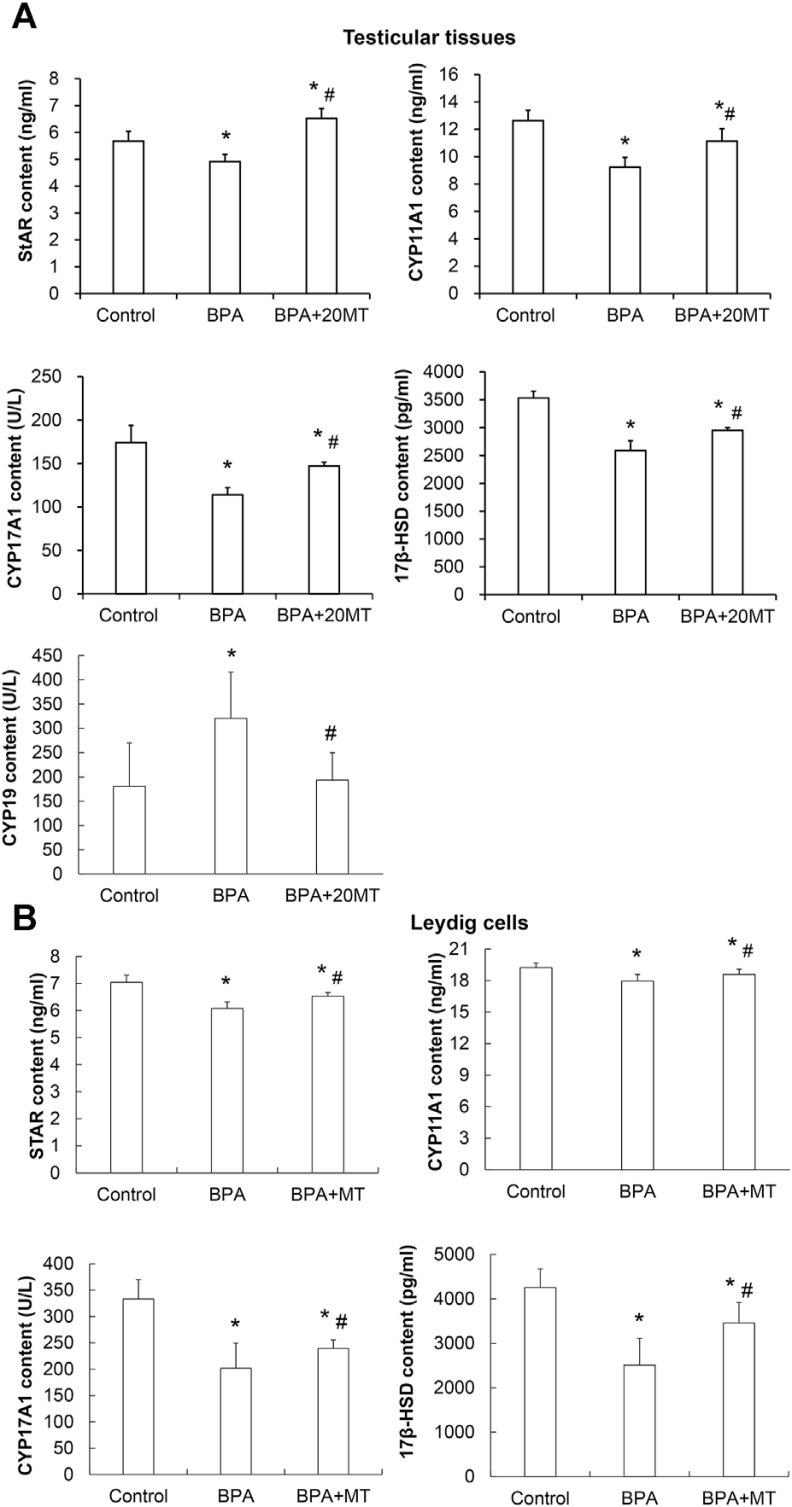
Expressions of steroid hormone synthesizing enzymes and aromatase in mouse testicular tissue and Leydig cells. **(A)** The expression of steroid hormone synthesizing enzymes StAR, CYP11A1, CYP17A1, 17β-HSD, and aromatase CYP19 in mouse testicular tissue was detected by ELISA (N = 5). **(B)** The expression of steroid hormone synthesizing enzymes StAR, CYP11A1, CYP17A1, and 17β-HSD in Leydig cells was detected by ELISA (N = 5). Compared with the control group, ^*^ *p* < 0.05; compared with the BPA group, ^#^ *p* < 0.05.

The expression levels of StAR, CYP11A1, CYP17A1, and 17β-HSD, which are steroid hormone synthesizing enzymes, in the testicular tissue and Leydig cells, as well as CYP19 aromatase in the testicular tissue, were detected by ELISA. The results revealed that in both the testicular tissue (Figure 8A) and Leydig cells (Figure 8B), the expression levels of StAR, CYP11A1, CYP17A1, and 17β-HSD in the BPA group were significantly lower than those in the control group (*p* < 0.05). However, their levels after melatonin treatment were significantly lower than those in the control group, but significantly higher than those in the BPA group (*p* < 0.05). Remarkably, the expression of aromatase CYP19 in the testicular tissue of the BPA group was significantly elevated compared to the control group (Figure 8A). In contrast, in the BPA+20MT group, the expression of CYP19 was notably lower than that in the BPA group and did not show a significant difference compared to the control group (Figure 8A). This data implies that melatonin could elevate the expression of steroid hormone-synthesizing enzymes in the testicular tissues and Leydig cells with BPA treatment, but lower the expression of aromatase in the BPA-treated testicular tissues.

### 3.8 Melatonin up-regulates the LHR expression and testosterone levels in the testes of BPA-treated mice and leydig cells and downregulates E2 expression in the BPA-treated testes

**FIGURE 9:**
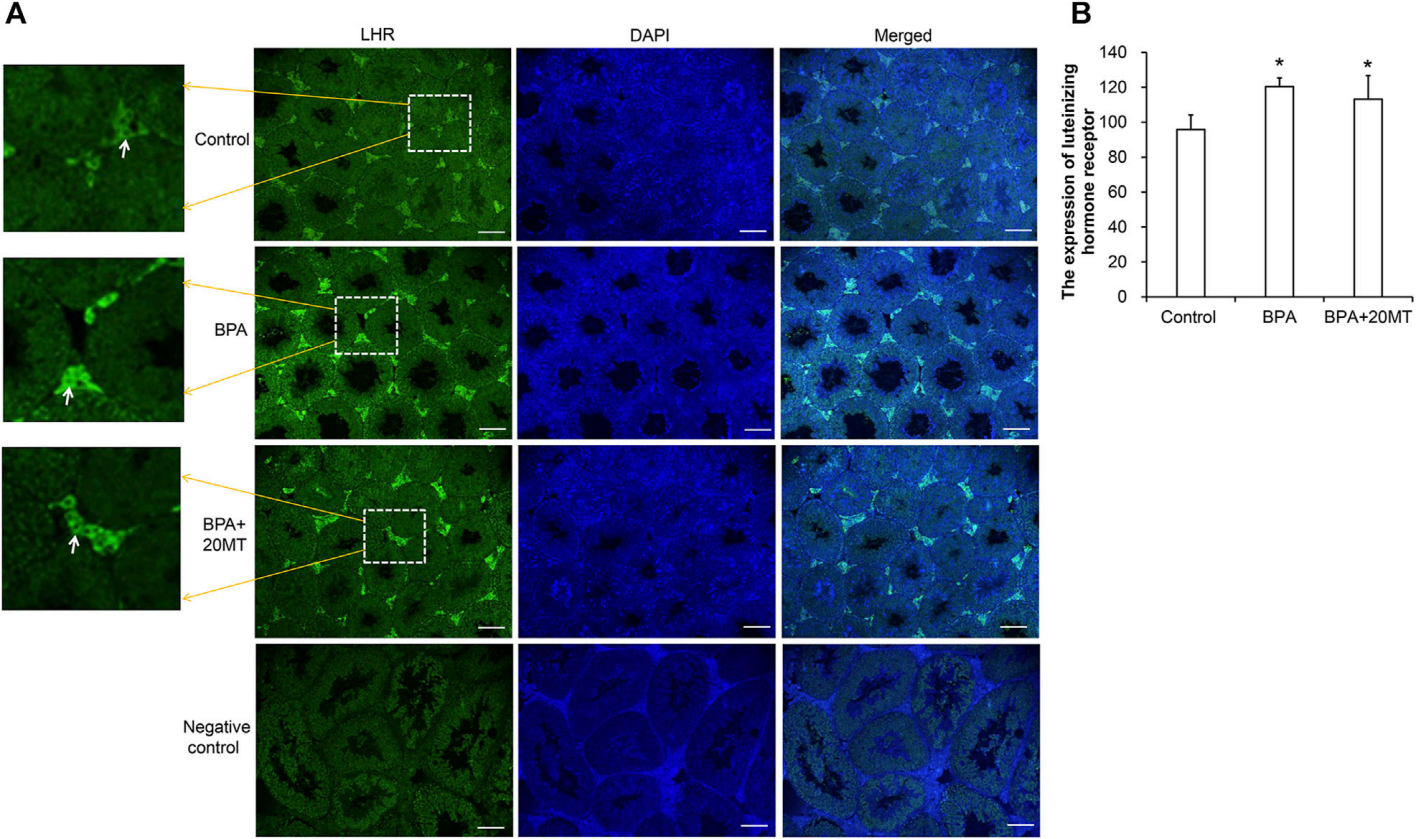
Detection of LHR expression in mouse testicular tissue. **(A)** LHR expression in mouse testicular tissue was detected using immunofluorescence. Green fluorescence represents the expression of LHR protein (Scale = 100 μm). The white arrow indicates the LHR expression. **(B)** LHR expression in each group of testicular tissue was represented by mean fluorescence intensity (N = 3). Compared with the control group, ^*^ *p* < 0.05.

The expression of LHR in mouse testicular tissue was detected using immunofluorescence, as shown in Figure 9. LHR was mainly expressed on the cell membrane of Leydig cells in the testes (Figure 9A). Compared to the control group, the BPA group and the BPA+20MT group showed enhanced green fluorescence in Leydig cells, suggesting increased expression of LHR. Statistically, the expression of LHR in Leydig cells was significantly higher in the BPA group and BPA+20MT group compared to the control group (*p* < 0.05) (Figure 9B). In contrast, there was no significant difference in LHR expression between the BPA group and the BPA+20MT group (*p* > 0.05). Thus, BPA promoted the expression of LHR on the cell membrane of Leydig cells, but the effect of melatonin on increased LHR expression induced by BPA was insignificant.

**FIGURE 10:**
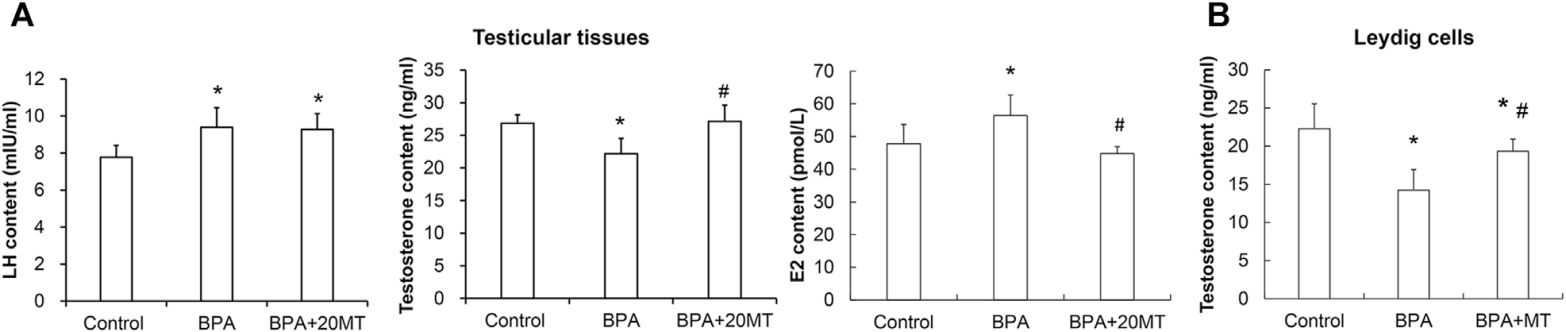
Levels of LH and E2 in mouse testicular tissue and levels of testosterone in mouse testicular tissue and Leydig cells. **(A)** The contents of LH, E2, and testosterone were measured in mouse testicular tissue using ELISA (N = 5). **(B)** The content of testosterone was measured in Leydig cells using ELISA (N = 5). Compared with the control group, ^*^ *p* < 0.05; compared with the BPA group, ^#^ *p* < 0.05.

The levels of LH and testosterone in mouse testicular tissue were measured using ELISA. As presented in Figure 10A, the levels of LH in the BPA group and BPA+20MT group were significantly higher than in the control group (*p* < 0.05). No significant difference in LH levels was found between the BPA group and the BPA+20MT group (*p* > 0.05). This data demonstrated that BPA increased the LH levels in testicular tissue, but the effect of melatonin on LH levels was not significant. Moreover, the testosterone levels in the testicular tissue of the BPA group were significantly lower than the control group (*p* < 0.05) (Figure 10A). Interestingly, the BPA+20MT group exhibited significantly higher testosterone levels than the BPA group (*p* < 0.05). The testosterone levels did not differ significantly between the BPA+20MT group and the control group (*p* < 0.05). Additionally, the testosterone levels in Leydig cells exhibited a consistent trend with those in the testicular tissue (Figure 10B). However, the level of E2 in the testicular tissue was significantly elevated in the BPA group compared to the control group but decreased significantly in the BPA+20MT group compared to the BPA group (Figure 10A). Additionally, there was no significant difference between the BPA+20MT group and the control group. These results suggest that melatonin facilitates the synthesis and secretion of testosterone while reducing the level of E2.

## 4 Discussion

Melatonin, as a potent antioxidant, can alleviate oxidative stress in various organs (Tordjman et al., 2017; Rehman et al., 2019; Wang et al., 2021; Xu et al., 2021). It has been clinically used for the treatment of autism (Lalanne et al., 2021), Alzheimer’s disease (Sumsuzzman et al., 2021), and endometriosis (Li et al., 2022). BPA is a commonly encountered endocrine disruptor in humans, which can cause oxidative stress damage in testicular tissues, leading to hormonal changes, reduced testicular weight, and impaired sperm production in mice (Behmanesh et al., 2018; Kabel et al., 2022). The use of antioxidants to alleviate testicular oxidative stress damage caused by BPA is an important measure for protecting the normal development of testicular cells. This study explored the effects of melatonin on the testicular tissues of male mice treated with BPA or BPA-treated Leydig cells, considering its strong anti-inflammatory and antioxidant properties. The results demonstrated that melatonin effectively alleviated oxidative stress-induced damage in testicular tissues or Leydig cells caused by BPA and protected the testes and epididymis.

BPA can increase the ROS and lipid peroxidation in testicular cells, and decrease the content of GSH as well as the expression of antioxidant enzymes SOD and CAT, leading to testicular cell apoptosis (Nguyen et al., 2022; Al-Griw et al., 2023). Taurine has been used to alleviate oxidative stress in mouse testicular cells (Rezaee-Tazangi et al., 2020). It has been found that semen cuscutae flavonoid can alleviate oxidative stress in testicular tissues of offspring caused by maternal/fetal transmission (Zhao et al., 2023), and glutamine can alleviate BPA-induced testicular toxicity by increasing the expression of CAT, SOD, GSH, and GSH peroxidase in testicular tissues (Ehigiator et al., 2022). Various antioxidants can alleviate oxidative stress in testicular tissues to different degrees. Melatonin, as a strong antioxidant, can alleviate H2O2-induced liver cell damage by regulating the extracellular regulated kinase/protein kinase B/nuclear factor-kappa B pathway (Moniruzzaman et al., 2018), mitigate oxidative stress damage in small intestine tissues caused by sepsis by upregulating the expression of sirtuin 3 (Xu et al., 2021), and attenuate oxidative stress damage in chondrocytes caused by H2O2 by regulating the AMPK/Foxo3 pathway (Chen et al., 2021). However, melatonin is rarely used to alleviate oxidative stress damage in testicular tissues caused by BPA. In our study, we found that melatonin resulted in a significant decrease in lipid peroxidation levels and a significant increase in the content of SOD1, SOD2, CAT, and GSH in the testicular tissues. These findings indicate that melatonin alleviates oxidative stress damage in BPA-induced testicular cells by enhancing their antioxidant capacity. Furthermore, melatonin significantly reduced the apoptosis of testicular cells induced by BPA. This suggests that melatonin can alleviate oxidative stress damage caused by toxic substances on testicular cells and has potential protective effects on testicular cell development.

Leydig cells in mammalian testicular tissue can synthesize and secrete testosterone, and high levels of testosterone are necessary for normal spermatogenesis (Chung et al., 2020). BPA reduces the expression of Leydig cell steroidogenic enzymes, including StAR, CYP11A1, CYP17A1, and 17β-HSD, thereby impacting testosterone synthesis and secretion (Zhang et al., 2023). Cholesterol is the precursor for testosterone synthesis, and it is transported by StAR to the inner mitochondrial membrane (Stocco, 2000). CYP11A1 catalyzes the conversion of cholesterol to pregnenolone on the inner mitochondrial membrane (Tremblay, 2015). Pregnenolone is converted to testosterone under the action of steroidogenic enzymes (such as CYP17A1 and 3β/17β-HSD) (Tremblay, 2015). Melatonin can promote testosterone synthesis and secretion by increasing the expression of steroidogenic enzymes such as StAR, steroidogenic factor 1, GATA binding factor 4, CYP11A1, and CYP17A1 in Leydig cells (Yang et al., 2021). However, it is unclear whether melatonin can increase testosterone levels in BPA-treated mice. In this study, melatonin was applied to BPA-treated male mice and BPA-treated Leydig cells, and it was found that melatonin significantly increased the expression of steroidogenic enzymes StAR, CYP11A1, CYP17A1, and 17β-HSD in the testicular tissue of BPA-treated mice and BPA-treated Leydig cells, thereby promoting testosterone synthesis and secretion. Testosterone increase could enhance the number of epithelial layers in the seminiferous tubules, promote the orderly arrangement of various stages of germ cells, and cause a significant increase in sperm viability and sperm density in the epididymal tail.

The synthesis of testosterone in testicular tissue is regulated by LH from the pituitary gland. Upon binding to receptors on Leydig cells, LH can activate adenylyl cyclase and G-proteins, thereby increasing the level of cyclic adenosine monophosphate (cAMP) (Jin et al., 2022). Elevated cAMP can enhance the activity of steroidogenic enzymes, facilitate cholesterol transport into the mitochondrial membrane, and promote testosterone synthesis. In young men, there is a significant positive correlation between serum LH levels and the concentration of BPA in urine and elevated LH levels could significantly decrease sperm count in semen (Adoamnei et al., 2018). BPA, as an endocrine disruptor, binds to G protein-coupled receptors 30 in testicular cells through a non-genomic signaling pathway, leading to rapid phosphorylation of protein kinase A and mitogen-activated protein kinases (Kitamura et al., 2005; Williams et al., 2014), affecting steroidogenesis in Leydig cells (Gerits et al., 2008; Barbagallo et al., 2020; Li et al., 2020), and decreasing the synthesis and secretion of testosterone in the testes. The hypothalamic-pituitary-gonadal axis has a negative feedback mechanism, such that when testosterone levels decrease in the male body, the pituitary gland is negatively regulated to secrete LH, while also increasing the expression of LHR on the interstitial cells of the testes for better LH regulation (Savchuk et al., 2013). In our study, we also found that there was a significant decrease in serum testosterone levels in BPA-treated mice or Leydig cells but a significant increase in LH and LHR in the testes of BPA-treated male mice. The regulation of the hypothalamic-pituitary-gonadal axis by melatonin is currently believed to be mediated through the medial basal hypothalamus region of the hypothalamus and the pituitary gland. Melatonin exerts its regulatory effects by binding to its specific membrane-bound receptors (MTNR1A, MTNR1B, and MTNR1C) (Talpur et al., 2018; Charif et al., 2022). MTNR1A is mainly distributed in the suprachiasmatic nucleus of the hypothalamus. The expression of MTNR1A suppresses the secretion of gonadotropin-releasing hormone, thereby inhibiting the release of LH and affecting animal reproductive function. In males, melatonin mainly exerts its effects by regulating gonadotropin-releasing hormone secretion from the hypothalamus and LH secretion from the pituitary gland (Diaz Lopez et al., 2005). However, we found that melatonin exhibited no significant effect on LH levels or LHR expression in the testes of BPA-treated mice. Considering the observed decline in testosterone secretion due to prolonged exposure to BPA in mice, the pituitary gland may react by compensatorily increasing LH synthesis and secretion, augmenting LHR expression in testicular cells, and intensifying the stimulatory influence of LH on testicular activity. Notably, when used as a therapeutic drug, melatonin exerts a regulatory effect on LH secretion (Perumal et al., 2020). However, in situations where BPA has already significantly raised LH levels and LHR expression in the testes of mice, the effect of melatonin on LH becomes less evident.

As an endocrine disruptor, BPA can affect the activity of aromatase in cells. Aromatase can catalyze the conversion of androstenedione and testosterone to estrone and E2 by removing the 19th carbon and aromatizing the A-ring, respectively, thereby disrupting the balance of estrogen and androgen, and leading to hormonal imbalance (Risalde et al., 2021; Sanchez et al., 2022). BPA has an affinity for the CYP19 gene (Faheem and Bhandari, 2021). The exact mechanism by which BPA acts on CYP19 is not fully understood. Faheem and Bhandari (2021) suggested that BPA disrupted the neuroendocrine regulation of reproduction by acting on the hypothalamic gonadotropin-releasing hormone, kisspeptin, and aromatase mRNA (Faheem and Bhandari, 2021); however, Molina et al. (2018) reported that low-dose BPA may have a direct negative effect on the transcriptional regulation of CYP19 expression in granulosa cells (Molina et al., 2018). In this study, we found that the expression of aromatase CYP19 in the testes of male mice treated with BPA was significantly increased, which can promote the conversion of testosterone to E2, leading to a significant decrease in testosterone levels and a significant increase in E2 levels. Melatonin can indirectly regulate estrogen synthesis by inhibiting aromatase activity (Jin et al., 2021) and it can also downregulate the expression of aromatase promoters II and I.3 (Bulun et al., 2005; Gonzalez et al., 2012; Knower et al., 2012). In addition, the activation of aromatase promoters II and I.3 is closely related to the elevated cAMP levels in epithelial cells through the secretion of prostaglandin E2 (PGE2) (Brueggemeier et al., 2003; Gonzalez-Gonzalez et al., 2019). The expression of PGE2 is regulated by cyclooxygenase. Melatonin can downregulate the activation of upstream cyclooxygenase-2 pathways, including ERK1/2, c-Jun N-terminal kinase, and p38 mitogen-activated protein kinases (Yao et al., 2019), reduce cyclooxygenase expression and PGE2 production, and decrease intracellular cAMP, resulting in reduced activation of promoters I.3 and II, decreased aromatase levels, and consequently reduced E2 levels (Martinez-Campa et al., 2009; Amin et al., 2019). In this study, we found that melatonin significantly reduced the elevated expression of aromatase CYP19 induced by BPA and significantly decreased the E2 levels in BPA-treated testicular tissues, playing an important regulatory role in restoring hormonal balance in testicular tissues. However, the specific regulatory mechanism still requires further study.

One important mechanism by which melatonin exerts its antioxidant effect is through binding to melatonin receptors on the cell membrane and activating antioxidant enzymes within the cell (Ferlazzo et al., 2020). Melatonin membrane receptors, specifically MTNR1A and MTNR2, are found in testicular and epididymal tissue (Zhang et al., 2022). Notably, MTNR1A gene polymorphism has been closely linked to various diseases, including oral cancer, polycystic ovary syndrome, type 2 diabetes, and Alzheimer’s disease (Lin et al., 2015; Sulkava et al., 2018; Yi et al., 2020; Amin and Gragnoli, 2023). Downregulation of MTNR1A can result in decreased testosterone levels within the testes, impacting normal physiological function (Gao et al., 2019). Moreover, drugs such as ethanol and acetaminophen have been shown to reduce the expression of MTNR1A (Adekeye and Fafure, 2022), thereby diminishing the regulatory effect of melatonin on tissue and cell development. Interestingly, melatonin itself is involved in regulating the expression of MTNR1A and has been found to play a role in mitigating anti-infection and anti-oxidative stress damage by inhibiting the loss of MTNR1A in amyotrophic lateral sclerosis (Zhang et al., 2013). Leydig cells are the primary cells expressing MTNR1A in the testicular tissue (Zhang et al., 2022). Our study found that BPA significantly reduced the expression of melatonin receptor MTNR1A. When melatonin and BPA act simultaneously on testicular cells, melatonin may exert its antioxidant effect by increasing the expression of MTNR1A on Leydig cells, thereby ameliorating oxidative stress damage to testicular cells, reducing cell apoptosis (particularly Leydig cells), promoting testosterone synthesis and secretion, and protecting testis and epididymis from BPA damage.

Oxidative stress can cause mitochondrial damage, ferroptosis, necrosis, and pyroptosis in cells. Notably, in testicular cells, mitochondrial damage caused by oxidative stress is a common cause of cell apoptosis (Karna et al., 2020; Chen et al., 2023). Mitochondrial oxidative stress can lead to an excessive generation of ROS that surpasses the scavenging capacity of the antioxidant system. These excess ROS can cause oxidation of thiol groups in mitochondrial membrane proteins, alter the permeability of the mitochondrial membrane, lead to the release of cytochrome C from the mitochondria, activate caspase 9, and induce cell apoptosis (Attardi, 2005). Damage to the integrity of mitochondrial membranes and the opening of the mitochondrial permeability transition pore are pivotal factors in the induction of the apoptosis pathway (Mohammadi-Bardbori and Ghazi-Khansari, 2008). Testicular spermatogenic cells have a high content of unsaturated fatty acids that are sensitive to redox reactions. Furthermore, due to the limited antioxidant content in the cytoplasm, these cells are highly susceptible to oxidative stress-induced damage (Koohsari et al., 2020). BPA can induce oxidative stress in testicular cells, increase the levels of ROS and MDA in mitochondria, significantly decrease the levels of SOD and GSH in mitochondria, activate the cell apoptosis pathway, and cause cell apoptosis (Rezaee-Tazangi et al., 2020). Our subsequent research also found that BPA significantly increased the levels of ROS in Leydig cell mitochondria, damaged the integrity of the mitochondrial membrane, reduced the mitochondrial membrane potential, disturbed the dynamic balance and biogenesis of the mitochondria, and impaired the function of the mitochondrial respiratory chain, which are important causes of cell apoptosis (data not yet published). Melatonin, due to its water-soluble and lipid-soluble properties, can easily permeate cell membranes, scavenge excess free radicals, and alleviate the occurrence of cell apoptosis (Xing et al., 2021). In addition, melatonin can also resist oxidative stress-induced cell apoptosis by binding to its receptor and activating the PI3K/AKT signaling pathway (Yang et al., 2020). This study found that melatonin significantly mitigated BPA-induced oxidative stress in testicular cells and reduced testicular cell apoptosis, but its specific mechanism of action still awaits further investigation.

After BPA expose, melatonin, as a potent antioxidant, can increase the expression of melatonin receptor MTNR1A on testicular cell membranes, enhance the content of antioxidant enzymes, reduce lipid peroxidation in testicular cells, decrease testicular cell apoptosis, improve testicular cell synthesis and secretion of testosterone, promote the normal development of seminiferous tubules, enhance semen quality, and play an important protective role in the function of the testes and epididymis. It holds great potential to become a critical pharmaceutical agent for the prevention and treatment of testicular oxidative stress damage, with widespread utilization in clinical practice.

## Data Availability

The raw data supporting the conclusion of this article will be made available by the authors, without undue reservation.
